# Improved Dynamic Light Scattering using an adaptive and statistically driven time resolved treatment of correlation data

**DOI:** 10.1038/s41598-019-50077-4

**Published:** 2019-09-18

**Authors:** Alexander V. Malm, Jason C. W. Corbett

**Affiliations:** Malvern Panalytical Ltd., Grovewood Rd, Malvern, Worcestershire WR14 1XZ UK

**Keywords:** Nanoparticles, Characterization and analytical techniques, Optical techniques

## Abstract

Dynamic Light Scattering (DLS) is a ubiquitous and non-invasive measurement for the characterization of nano- and micro-scale particles in dispersion. The sixth power relationship between scattered intensity and particle radius is simultaneously a primary advantage whilst rendering the technique sensitive to unwanted size fractions from unclean lab-ware, dust and aggregated & dynamically aggregating sample, for example. This can make sample preparation iterative, challenging and time consuming and often requires the use of data filtering methods that leave an inaccurate estimate of the steady state size fraction and may provide no knowledge to the user of the presence of the transient fractions. A revolutionary new approach to DLS measurement and data analysis is presented whereby the statistical variance of a series of individually analysed, extremely short sub-measurements is used to classify data as steady-state or transient. Crucially, all sub-measurements are reported, and no data are rejected, providing a precise and accurate measurement of both the steady state and transient size fractions. We demonstrate that this approach deals intrinsically and seamlessly with the transition from a stable dispersion to the partially- and fully-aggregated cases and results in an attendant improvement in DLS precision due to the shorter sub measurement length and the classification process used.

## Introduction

Dynamic Light Scattering (DLS)^1,2^, otherwise known as Photon Correlation Spectroscopy (PCS) or Quasi-Elastic Light Scattering (QELS), is a light scattering technique widely used to characterise nanoparticle systems^3^ in dispersion, given its sensitivity to small and low scattering cross-section particles, relative ease of use and comparative low capital outlay. Whilst electron microscopy is limited in applicability to samples with an appropriate electron-scattering cross section^4^, and requires the sample to be dried or more recently confined to a costly and specialised sample presentation system, DLS *directly* characterises particles in dispersion, and allows monitoring of dispersion effects such as; the effect of salt or pH on colloidal stability; the impact of thermal or chemical stresses on protein denaturation; or the solvation of a nanoparticle^5–7^, as well as facilitating measurements of parameters such as aggregation temperature *T*_*agg*_ and the diffusion interaction parameter *k*_*D*_, amongst others.

**Figure 1:**
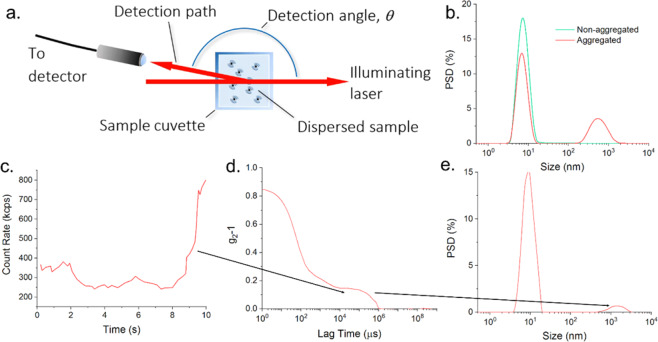
(**a**) Schematic of a typical DLS instrument, configured to detect backscattered light from the dispersed sample. (**b**) Replicate time averaged particle size distributions for a protein sample, showing the intermittent appearance of a large size component which also coincides with a perturbation to the position of the primary peak. (**c**–**e**) Analysis process of a DLS measurement- Light scattering intensity (**c**) showing a spike in the signal due to the appearance of an aggregate at t > 8 s, which can which can degrade the measured correlation function (*g*_2_-1) (**d**) and the accuracy of the intensity weighted particle size distribution (**e**).

A schematic of a typical DLS instrument is shown Fig. 1a, with an illuminating laser and single-mode fibre detection path from the cuvette to the detector described by the ***q***-vector,

1 $$|{\boldsymbol{q}}|=\frac{4\pi n}{\lambda }sin(\frac{\theta }{2})$$

where, *λ* is the wavelength of the illuminating beam in air, *n* is the refractive index of the liquid phase of the dispersion and *θ* the angle, in free space between the laser and the detection path.

In most commercial DLS instruments, the sample is presented in a simple disposable cuvette, with measurement times of typically a few minutes in comparison to time consuming separation based techniques such as size exclusion chromatography (SEC)^8^ and analytical ultra-centrifugation, (AUC)^9^. DLS also analyses a statistically superior number of particles in comparison to electron microscopy^10^ and is able to detect a wider particle size range than nanoparticle tracking analysis, NTA^11,12^ resulting in numerous important applications, including those of contemporary note such as manufacturing, food^13^, medicine^14,15^, environmental science and for screening and characterisation in biopharmaceutical development^16–18^.

The light scattered from many samples appropriate for characterisation by DLS is well approximated by the Rayleigh scattering model where the scattered intensity, *I*, Eq. 2, is proportional to the sixth power of the particle radius, *r*, for fixed wavelength, *λ*,

2 $$I\propto \frac{{r}^{6}}{{\lambda }^{4}}$$

and DLS draws criticism for its sensitivity to even very low concentrations of contaminant size fractions such as filter spoil, dust from improperly cleaned lab-ware and aggregated or unstable and aggregating samples which dominate the component of interest, thereby skewing a distribution based result such as a non-negative least-squares (NNLS) reduction^19^ as shown in Fig. 1b, or the *Z*_*Ave*_ cumulant^20^, the size average across all size classes.

Schemes to mitigate these effects might include the measurement of the sample over very long correlation times so that transient scattering from low concentrations of aggregated material, for example, is effectively averaged out of the overall result^19,21^ or sufficiently detected to be incorporated into a fitting model^22,23^; or data *rejection* methods such as the exclusion of aggregated, short sub-measurements, from the final averaged result^24–26^, typically based upon count rate/scattering intensity. Sub measurement durations for DLS instruments of the order of 10 s are common, Fig. 1c.

Deselection of data based on the scattered intensity may appear sensible at first glance, however, in practice, a deselection threshold would need to be pre-set for every different sample and sample type based on *a priori* knowledge of the sample concentration and particle size distribution, both of which contribute to the overall scattering level. This process is therefore not statistically relevant with respect to each *unknown sample* being measured and *these schemes can therefore result in a serious reduction in the efficiency of the measurement via significantly increased measurement times with, potentially, no attendant improvement in the signal to noise; or worse still, the possible skewing of the result by the removal of data from the aggregated fractions without the operators’ knowledge and finally, the desensitisation of the whole measurement to genuinely trending samples*.

## Development of a New Approach to DLS

In this work we describe and assess a new DLS measurement and data treatment process which cuts the photon arrival time data from the detector into very small blocks, each of which is correlated as a separate sub-measurement. The statistical distribution of a number of quantities derived from each correlated sub-measurement, built up during the measurement process, is then used to classify transient events, such as the one shown at the end of the 10 s sub-measurement, between 8 s and 10 s in Fig. 1b, and to analyse them separately to the remaining steady state data (0 s to <8 s in Fig. 1c). The result is then separately summed as a transient and steady-state correlogram pair, which are then reduced to yield the transient and steady-state particle size distributions. Crucially, since all of the collected data (transient and steady state) are analysed and reported: no data are rejected or hidden from the user and *a complete and un-skewed representation of any sample results, polydisperse or otherwise, but without the increased uncertainties in the steady-state fractions of interest in the presence of strong transient scatterers*. Further, this process deals intrinsically with the limiting case where so many aggregates exist that the primary fraction of the sample should be considered to be these larger components, i.e. the aggregates become so numerous that their signal *becomes* the steady-state fraction^27^.

We also find that the classification and separate reduction of the transient and steady state classes based on very short measurement sub-runs and in a manner *based on the statistics of the data themselves*, leads to a statistically relevant minimisation of the variability within the steady state class over short total measurement times leading directly to *an increase in the precision of the steady state DLS data* whilst simultaneously *reducing the total measurement time* for a well behaved sample, by an order of magnitude over those currently found in commercially available instruments.

The development of the technique is described in the remainder of this section using measurements of polystyrene latex particles as a model system of known sizes and dispersions of lysozyme as a fragile, low scattering sample. A number of case studies demonstrating the benefits of the technique are described in Section 3 with conclusions drawn in Section 4 and the methods used, described in Section 5.

### Analysis methods

Whilst the technique is equally applicable to cross-correlated measurements, most commercially available instruments measure the autocorrelation function *g*^2^(|***q***|;*τ*) of the detected, scattered photon time series *I(t)* given by,

3 $${g}^{2}(|{\boldsymbol{q}}|;\tau )=\frac{\langle I(t)I(t+\tau )\rangle }{\langle I(t){\rangle }^{2}}$$

where *τ* is the delay time, and *I* the measured intensity at the detector in photon counts per second measured at time *t*. The first order correlation function, *g*^1^, is retrieved from *g*^2^ via the Siegert relation^1^ and a cumulants-fit^20^ to *g*^1^ is commonly implemented such that,

4 $${g}^{1}(|{\boldsymbol{q}}|;\tau )=exp(-\bar{\Gamma }(\tau -\frac{{\mu }_{2}}{2!}{\tau }^{2}+\frac{{\mu }_{3}}{3!}{\tau }^{3}+\ldots ))$$

where, $\bar{\Gamma }\,$ is the average, characteristic decay rate over all size-classes in the sample and ${\mu }_{2}/{\bar{\Gamma }}^{2}\,$is the 2^nd^ order polydispersity index (*PdI*) which describes the departure of the correlation function from a single exponential decay providing an estimate of the variance of the sample. The *z*-average diffusion co-efficient, *D*_*z*_, is then given by the relation

5 $$\bar{\Gamma }={|{\boldsymbol{q}}|}^{2}{D}_{z}$$

and the average hydrodynamic diameter, *Z*_*Ave*_, calculated from *D*_*z*_, using the Stokes-Einstein model for spherical particles in liquids with low Reynolds number, Eq. 6, where *η* is the dispersant viscosity, *k*_*B*_, the Boltzmann constant and *T*, the dispersant temperature in Kelvin,

6 $${D}_{z}=\frac{{k}_{B}T}{3\pi \eta {Z}_{Ave}}$$

An estimate of particle size distribution to higher resolution than cumulants is given by fitting the correlation function to a sum over multiple exponentials, accomplished by a number of possible inversion methods such as CONTIN^28^ or non-negative least squares (NNLS), which are two commonly implemented examples designed to cope with the generally ill-posed nature of such a fit. For the polydisperse case, Eq. 4 then becomes a continuous distribution over *D*, from which a range of contributing particle radii or diameters can be calculated,

7 $${g}^{1}(|{\boldsymbol{q}}|;\tau )=\int G(\Gamma )\,exp(\,-\,\Gamma {\rm{\tau }})d\Gamma $$

### Sub measurement length and improved precision

The photon arrival time series is divided into small sub measurements which are then individually correlated and reduced into sample properties as described in Section 2.1 and distributions of these derived quantities, built up as the measurement proceeds, are then used to identify *transient* and *steady-state* data.

The experimental uncertainty in quantities derived from the DLS data (*Z*_*Ave*_, *PdI*, count-rate, etc.) over multiple measurements is inversely proportional to the square root of the number of measurements in the usual manner, however, the relationship between the noise in the correlogram within each sub measurement and the sub measurement length is less obvious. Recalling Fig. 1a, the sampled volume; the region subtended by the intersection of the illuminating laser and the detection paths, both of finite width; is significantly smaller than the total volume of sample in the cuvette, therefore, as the integration time increases the probability that an aggregate diffuses into, or out of, the detection volume increases and in this section is to explore how the derived quantities, *Z*_*Ave*_ and *PdI* behave as a function of the sub measurement duration. The aim is to optimise the duration in order to maintain or improve the signal-to-noise, but in a sub measurement length that simultaneously allows the selection algorithm to remain responsive enough to classify each sub measurement as steady state or transient.

**Figure 2:**
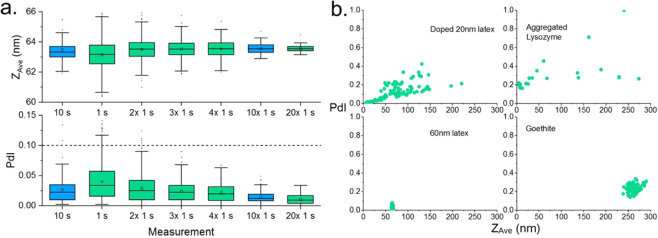
(**a**) Distribution of *Z*_*Ave*_ and *PdI* as a function of sub measurement duration and number of sub measurements. All recorded data are shown. i.e. no data were de-selected for this figure: See main text for discussion. The dashed line shows the ISO standard for polydispersity index. (**b**) Examples of polydispersity index, *PdI*, as a function of *Z*_*Ave*_ for samples containing trace amounts of additional large material (top), (see supplementary information) and stable, well-prepared samples (bottom).

Figure 2a shows the *Z*_*Ave*_ and *PdI* for a series of measurements of a polystyrene latex with a hydrodynamic size range specified by the manufacturer as 58–68 nm (Thermo-Scientific, 3060 A), dispersed in 150 mM NaCl made with 200 nm filtered DI water (18.2 MΩ).

Note the reduction in the standard deviation over the measured *Z*_*Ave*_ from 1.1 nm to 0.32 nm between the 1 × 10 s and 10 × 1 s cases, highlighted in blue indicating that *the precision of the DLS measurement is increased simply using an average over shorter sub-measurements but for the same total integration time*. Similar behaviour can be seen in measurements of different sized particles (see the Supplementary information).

The mechanism behind this improvement can be explained by considering the form of the correlation function when a transient scatterer is detected. The correlation function is approximately an exponential decay, with small perturbations due to several sources of noise including after pulsing, shot noise, normalisation errors and of course the detection of differently sized scattering particles^21^. Recording the correlated light scattering over short time intervals may increase the amplitude of these perturbations, but averaging over several sub measurement correlation functions, each containing random noise, means that the final result contains less noise than a correlation function recorded with the same duration but treated as one continuous trace. *This is an extremely important result as it indicates that nothing more than a carefully derived sub-measurement length yields a 3-fold improvement in the precision for this primary nanoscale measurement modality*.

Further, as we show in the next section, the shorter sub measurement length also allows the classification of steady-state and transient data, which we will demonstrate solves a primary criticism of DLS: the proportionality of the scattered intensity to the sixth power of the particle radius, meaning that data from the primary particle component may be skewed or even masked by the presence of rare large particles. In practical terms, this imposes the necessity of scrupulous sample preparation to avoid significant uncertainties in the outcome of the measurement caused by larger, often unwanted fractions, from filter spoil, transient aggregates or poorly cleaned lab-ware, for example.

### Classification of transient and steady state data

As stated previously, many commercial DLS instruments use a sub measurement time of the order of 10 seconds with several of these measurements being combined following some form of dust rejection algorithm, however means that large sections of reliable data may be omitted from a measurement if a sub measurement contains a short burst of scattering from a transient event. This hints that a classification of steady state and transient data might also be achieved by using shorter correlation times and this may also make comparison between sub measurements more accurate as the effects of transient scattering would not be averaged out. The results of a series of these sub measurements could then be combined by analysing the average of the autocorrelation functions before performing size analysis, as discussed in Section 2.2.

All recorded sub measurements are then classified into sets that describe the steady state and transiency of the system or in other words those that are a representative of the underlying, steady state sample and those that are associated with a burst of spurious scattering as shown in Fig. 1c.

The identification of transient sub measurements should be derived from the characteristics of the sample under investigation to avoid the need for arbitrarily defined thresholds that may be sample specific. By reducing each of the collated sub measurements individually, a number of possible parameters are available which may be used as the basis for comparison of sets of sub measurements and it seems logical to base this comparison on a size analysis of the measured autocorrelation functions.

Cumulants analysis assumes that a sample is monodispersed, meaning that both the *Z*_*Ave*_ and *PdI* will both give continuous and sensitive measures of the particle size that we can use to compare sub measurements. The *PdI* describes the departure of the correlation function from a perfect exponential decay. It is a direct measurement of the perturbation of the correlation function and is especially sensitive to noise in the correlation function baseline which is a typical consequence of transient scattering and, as we will show, is therefore an ideal parameter to use to compare the correlation functions from a plurality of sub measurements.

An example of such a relationship is shown in Fig. 2b, where the samples contain either aggregated material or are doped with a mixture of latex spheres (see supplementary information). Here, samples containing trace amounts of aggregate show a positive correlation between measured size and *PdI*, with some data points clustered at a consistent size and *PdI*, whereas un-doped samples show well defined clusters of data. Transient sub measurements can therefore be identified as those that present at an unexpected value for the *PdI*. In this instance, unexpected means that the *PdI* of a given sub measurement is not representative of the steady state sub measurements and is therefore a statistical outlier. Many methods exist for identifying statistical outliers, each having strengths and weaknesses depending on the nature of the distribution of interest and the sample size.

**Figure 3:**
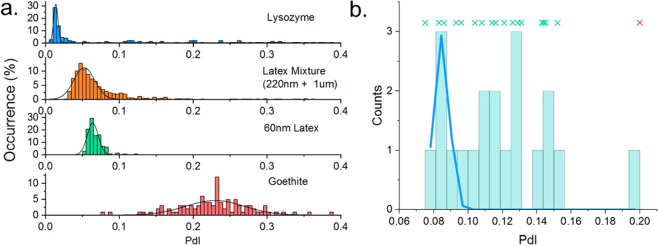
(**a**) Distributions of PdI for a range of aggregated/contaminated samples, demonstrating the need for a sample specific definition to identify the measurement of transient particles. These distributions also show that the PdI is a biased distribution, and as such, a three standard-deviations from the mean threshold for outliers would not be robust. (**b**) Histogram of a sparsely collected set of measurements for a sample of lysozyme. Whilst fitting using a least squares regression and a Gaussian model in (**a**) reliably allowed the statistics of sufficiently sampled data sets to be determined, an attempt to fit to a sparse data set is shown in blue but shows poor correlation with the distribution data due to apparent under sampling. Also shown is a scatter plot of the individual values showing their spread. The individual point show in red is successfully identified as an outlier by the Rosner generalised many outlier procedure.

Figure 3a shows the distributions of the *PdI* for dispersions containing arbitrarily small amounts of spurious material, with distributions of the *PdI* varying in centre and width for the different samples. Given that *PdI* is limited, by definition, to the interval [0.00,1.00] and will generally be skewed towards larger values, arithmetic descriptors of the distribution such as the mean and standard deviation are not appropriate.

Where the number of discrete sub measurements is sufficiently large a histogram of the data may be used to derive a distribution width (see Gaussian fits in Fig. 3a), however when the sample size is smaller, numerical hypothesis test methods such as those described by Dixon^29^ and Rosner^30^ may be more appropriate, Fig. 3b.

### Optimising sample size

The efficiency of any outlier identification method will be coupled to both the total number of data points and the number of outliers within a distribution. For example, the well prepared, monodisperse and stable sample shown in Fig. 2a demonstrates that a reliable size can be reported for as few as 10 averaged sub measurements of 1 s duration, whereas a sample that produces more noisy correlation functions, either through low scattering, having significant polydispersity or by containing spurious scatterers, will require a greater number of sub measurements in order to give more confidence to the identification of outliers. Again, this motivates a sample driven approach whereby the number of sub measurements is responsive to the quality of the data gathered from the sample.

Possible approaches might be to monitor the spread of the individual sub measurement results or to perform tests for normality on these values, however this would typically drive the measurement to acquire a greater number of data points. An alternative approach is to continuously monitor the would-be final result as the measurement progresses, where the statistics of the measurement are suitably well defined, and the perturbations in the correlation function are suitably well averaged out of the final result, the reported size should become constant to within some degree of natural variation. Again, hypothesis tests can be used to compare the would be result of the measurement after the gathering of additional sub measurements, and if these values agree, then the sample is adequately characterised, and the measurement can end accordingly. Further confidence can be added to this method by checking for special causes in the results throughout the measurement, such as trending and oscillation.

**Figure 4:**
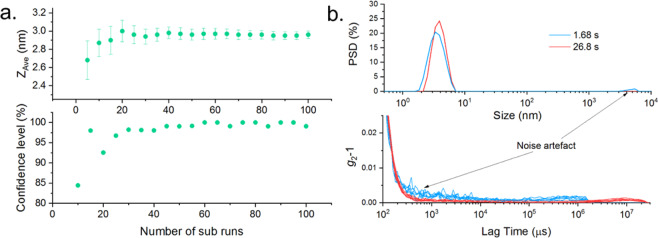
(**a**) Top: The reported *Z*_*Ave*_ against the number of measured sub measurements during the measurement of a sample of lysozyme. An estimate of the standard error on each reported size is shown by error bars. The result is initially inaccurate and variable but stabilises after a sufficient amount of data is gathered. Bottom: Confidence level (CL) of a hypothesis test of data similarity, calculated for successive values shown for the *Z*_*Ave*_. When the confidence level has reached a threshold, no resolvable difference in *Z*_*Ave*_ is expected and recording of additional sub measurements can therefore end. (**b**) Top: Intensity weighted particle size distribution for measurements of 1 mg/mL lysozyme using short and long correlation times measured at a 90° detection angle. The short sub measurements show an apparent large size component which is a noise artefact associated with the low scattering intensity of the sample. Bottom: Corresponding correlation function baselines for repeat measurements using long and short sub measurements. The short sub measurements show a temporally resolved, additional decay artefact.

An example of this approach is shown in Fig. 4a for a sample of lysozyme, with an initially erroneous underestimation of the particle size reported, but which stabilises with the collection of subsequent sub measurements. Note also that outlier identification is repeated during the measurement as more data is gathered, meaning that a transient event will be identified as such, regardless of when in the measurement process it was recorded. This is an improvement on other methods that may compare data based on an initial measurement, which may or may not have been representative of the true sample.

This results in an improved data gathering efficiency without user intervention and measurements of stable samples that require less data to be gathered can therefore be completed in a shorter time, whereas complex samples that show some level of uncertainty will automatically have a greater amount of data gathered to yield a result with comparable confidence.

### Sampling optimisation

As described in Section 2.2, there are several sources of noise in the correlation function and the amplitude of this noise can be temporally dependent. While Section 2.2 made the case for using short correlation times, there are instances where this may be detrimental.

For a sample that demonstrates low scattering properties, through either a small scattering cross section, low sample concentration, small difference in refractive index to the surrounding dispersant or a combination thereof, there may be fewer detected photons to populate the correlator time bins and this will typically manifest itself as noise in the correlation function baseline at longer correlator delay times, *τ*.

Commercial light scattering instruments typically vary a number of instrumental settings as part of the measurement set-up procedure, such as the optimisation of the measurement position within the cuvette to minimise the optical path length of the ingoing laser and the outgoing scattering detection path to avoid multiple scattering from concentrated samples near to the cuvette centre or, conversely, to avoid static scattering from the cell wall at low sample concentrations and the optimisation of the detected photon count-rate to stay within the linear range of the detector. These instrumental optimisations are generally designed to allow users that are unfamiliar with interpreting light scattering data to retrieve the most reliable results over a broad range of sample concentrations and sizes, but such optimisation has not previously been applied to the correlation time. An example of this is shown in Fig. 4b, with particle size distributions shown for a sample of 1.0 mg/mL lysozyme measured at a 90° detection angle. The reported PSD for a short correlation time shows an apparent large size component in addition to the main particle peak.). If this was a real sample effect, measurements at a smaller detection angle would have shown the same large component. The forward scatter measurements for the same sample were monomodal (see SI) and the absence of the peak at in the measured data at other detection angles (Supplementary information) indicates that it may have been due to a combination of a low scattering sample and static scattering, possibly, from the disposable sample cuvette. Whilst the intensity of the incident light may be optimised, some samples such as low concentration proteins may scatter a sub optimal number of photons even with no attenuation of the illuminating laser, meaning that standard operating processes for a commercial dynamic light scattering system may not be optimal and longer correlation times may be used^24^ with extensive method development required to determine these settings. A further sample driven adaption of the measurement can therefore be introduced, whereby the instrument uses the shortest possible sub measurement length that will yield an optimum number of photons to be measured (see SI) and this is described in the optimised measurement scheme in the next section.

### The optimum measurement scheme

The optimum measurement scheme is made up of the following process:

1. Optimisation of the measurement position and incident light intensity.
2. If the detected scattering level is low even with the lowest laser attenuation, the sub measurement length is optimised to reduce baseline noise.
3. Sub measurements are gathered and analysed using cumulants analysis.
4. The *PdI* values from these analyses are compared and outliers identified.
5. The correlation functions of the steady state sub measurements are averaged, and the result analysed to report a *Z*_*Ave*_.
6. More sub measurements are recorded and analysed as above, and a new final answer *Z*_*Ave*_ recorded.
7. This process is repeated until the previous two *Z*_*Ave*_ results from steps (5) and (6) are found to agree using a hypothesis test.
8. All transient sub measurements are also averaged and analysed to provide information on the transient component.

Given the response of the above algorithm to the sample characteristics, with sub measurement length, amount of data collected and which sub measurements to omit from the steady state result all being sample and data quality dependent, the method is dubbed Adaptive Correlation, taking inspiration from the use of Adaptive Optics in astronomy^31^, where data feedback is used to correct for observed aberrations.

## Results and Discussion

Due to their typically low scattering cross section and a tendency to naturally aggregate, particle sizing of proteins is particularly challenging^32^ and we demonstrate the improved characterisation of this important class of samples using Adaptive Correlation in this section. A sample of lysozyme was prepared to a concentration of 5.0 mg/mL in an acetate buffer and filtered using syringe filters (Whatman Anotop, 10 mm diameter) of different pore sizes before being measured using Adaptive Correlation and an alternative DLS data processing method that uses 10 s sub measurements and data rejection based on threshold count rates.

**Figure 5:**
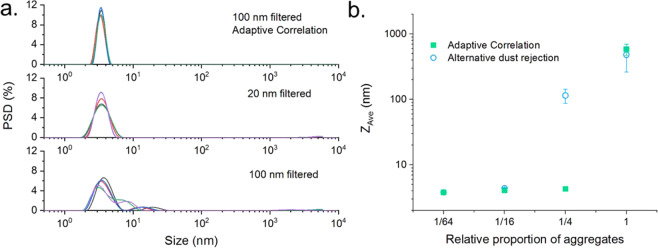
(**a**) Intensity weighted particle size distributions for a 5 mg/mL dispersion of lysozyme. The top figure was generated using Adaptive Correlation, with an aliquot filtered using a 100 nm filter. The middle and bottom figures show results using a legacy method, with the sample filtered using a 20 nm and 100 nm filter respectively. (**b**) Reported *Z*_*Ave*_ for measurements of the size of lysozyme, following different filtering processes. Results are shown for Adaptive Correlation and a previously used dust rejection technique. A sharp inflection in the data is seen as the average size becomes dominated by the presence of a small mass of larger aggregate particles.

Figure 5a shows that when filtered through a 100 nm filter, Adaptive Correlation produces repeatable monomodal particle size distributions, whereas the data gathered without Adaptive Correlation shows a larger size component. In fact, despite an improved measurement quality, even the 20 nm filtered data does not reproduce the adaptive correlation result. This shows that good quality and reliable particle sizing results can be obtained using Adaptive Correlation that may not otherwise be generated even with the use of more costly, smaller pored filters. Additionally, the ability to use larger pore filters facilitates less laborious sample preparation, as larger filters can generally be used to filter greater volumes, are less likely to become blocked and exert lower shear forces on the sample.

The reduction in sensitivity of measurements to the presence of aggregates can also be demonstrated by measuring aliquots containing varied amounts of aggregates and the data shown for the average particle size, *Z*_*Ave*_, in Fig. 5b was gathered by first measuring a sample of unfiltered lysozyme, and then removing half of the sample and then filtering this removed volume back into the cuvette. This was repeated until no change in sample quality could be observed and whilst this does not represent a real-life sample preparation procedure, it serves to generate a concentration ladder in which each sample contained half the number of aggregates compared to the previous aliquot. Whilst filtering of the sample will inherently lead to some reduction in sample concentration, UV absorption measurements showed that the final concentration was within 10% of the initial concentration. The inflection in the data in Fig. 5b shows that at higher concentrations of contaminant, the reported average particle size is dominated by the small quantities of the much larger material. Adaptive Correlation is therefore shown to report an accurate *Z*_*Ave*_ for samples containing approximately 16 times the amount of aggregates that can be tolerated by a previously used dust rejection scheme. In terms of absolute value for the tolerable concentration of contaminants, this is highly sample and measurement specific and will depend on factors including but not limited to the primary particle size, concentration, refractive index of both the dispersed material and the dispersant, the dispersant viscosity and the scattering detection angle. As an example, for lysozyme prepared in an aqueous buffer, the tolerable concentration of contaminants was estimated based on dilution and doping from a known concentration measured at stock concentration of a monomeric and aggregated suspension. For a 5 mg/ml sample of lysozyme, measured in a backscatter configuration, the steady state result was reliably reported as monomeric when the ratio of monomer to aggregate particles (d ~ 500 nm) was 10^10^:1, however the same sample measured at a 90-degree scattering angle did show aggregated components present in the steady state result, SI Figure S7. An aggregate component was also detected in the steady state result for a similar backscatter measurement for a 2 mg/ml sample of lysozyme in the same buffer conditions and with the same monomer-aggregate ratio of 10^10^:1, demonstrating that there is no universal tolerable limit for contamination.

**Figure 6:**
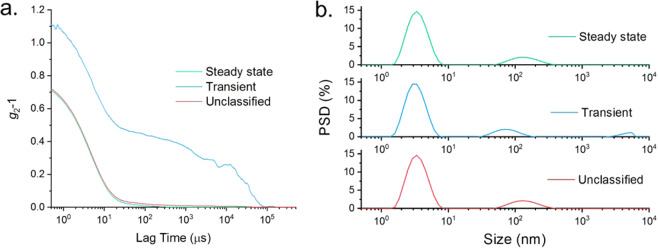
(**a**) Autocorrelation functions for the steady state, transient and unclassified data for a measurement of 5 mg/mL dispersion of lysozyme. The unclassified and steady state data appear closely comparable, but analysis of the transient data gives insight to the presence of trace large particles. (**b**) Intensity weighted particle size distributions for an aggregated sample of 5 mg/mL lysozyme, calculated independently for the steady state, transient and unclassified data sets. In all instances, a monomer and aggregate peak are observed at 3.8 and ~100 nm, however the transient data also shows an additional large size peak.

Whilst it has been shown that Adaptive Correlation can provide a more robust results for particle sizes with reduced sensitivity to large spurious material, there are many applications where the detection of aggregates is of critical interest. By classifying sub measurements into transient and steady state data sets, Adaptive Correlation gives both an improvement in the precision of the steady state result but also better insight to the transient data as *no data are rejected*. Of additional benefit is that the analysis of the transient material may be reported to a higher resolution as this information may be averaged out and not properly resolved for more traditional, sum-over-all-data reductions. Figure 6a demonstrates this with a comparison of the correlation functions for the steady state and unclassified cases (i.e. results included from all sub measurements), which show only a minor difference in the baseline, however the transient correlation function shows a significant additional decay.

Figure 6b demonstrates that the Adaptive Correlation approach is not a filter on size. The result shows the presence of aggregates at approximately 100 nm, present in the unclassified data. The steady state data show a comparable distribution, but the transient data shows a size component that was not resolved in either of the other distributions as the contribution of this size population was averaged out over the duration of the whole measurement. This clearly demonstrates that adaptive correlation sidesteps the pathological effects of large transient scatterers on DLS data due to the sixth order proportionality between scattered intensity and the particle size. Further still, by quantifying the detection of transient scatterers we can also derive additional information regarding the stability of a sample that may otherwise appear stable. As demonstrated, the identification of transient particles is an adaptive process meaning that the number of transient points will vary and can be zero. By tracking the retention rate, i.e. what percentage of sub measurements in a measurement were identified as steady state, underlying trends can be observed, as shown in Table 1.

**Table 1:** Size results measured for trending sample of lysozyme, showing comparable *Z*_*Ave*_ and Peak size results, but a decreasing sub measurement retention rate, indicating the onset of aggregation.

Z_Ave_ (nm)	*PdI*	Peak One Size (weighted by Intensity) (nm)	Retention Rate (%)
3.90	0.14	4.59	100
3.87	0.13	4.52	100
3.95	0.16	4.69	100
3.77	0.20	4.91	97
3.77	0.22	5.07	96
3.85	0.15	4.57	80
3.84	0.15	4.60	75

The data presented in Table 1 for a sample of lysozyme measured at 30-minute intervals shows that while the steady-state component of the sample is monomodal, with only one peak observed in the distribution and no apparent trend in either *Z*_*Ave*_, *PdI* or peak size, the measurement retention decreases indicating that aggregates are being detected with increased abundance over time. For example, this approach could be used to screen dispersants or formulations with different preparations of a sample potentially giving comparable size results, but one formulation presenting a higher probability of aggregates being detected in small numbers.

In order to demonstrate the applicability of analysing the transient data, aliquots containing two different sizes of polystyrene latex, 60 nm and 1.6 µm, were prepared with different ratios of the two sizes.

**Figure 7:**
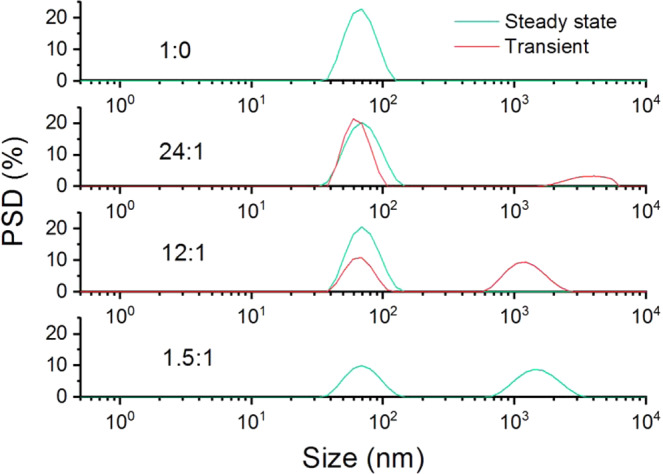
Intensity weighted particle size distributions for polystyrene latex mixtures, containing 60 nm latex and 1.6 µm latex at different volume ratios, measured at a 90° scattering angle. With increasing concentration of the large size component, a transition is seen between this not being detected, appearing in only the transient data and then in the steady state.

Figure 7 shows a transition between a monomodal result and a bimodal result being reported for the steady state. In each of these cases, no sub measurements were identified as being transient. At intermediate concentration ratios for the two particle sizes, some sub measurements were identified as transient and an additional size peak is reported in the distribution result.

When the concentration of the 1.6 μm latex becomes sufficiently high, the transient data become resolvable with an accurate size peak being reported for the transient component of the sample: in this case, 12:1. Whilst this demonstrates the effects of the adaptive correlation approach in correctly handling transient data within the reportable size range for a typical DLS measurement, transient particles may be larger than the 10 µm size limit displayed in the distribution results discussed in this manuscript, and it has been shown that reliable particle size results for the primary component may be gathered in the presence of much larger particles that display significant slow modes beyond the distribution analysis range, as distribution results are not used in the classification of transient events.

**Figure 8:**
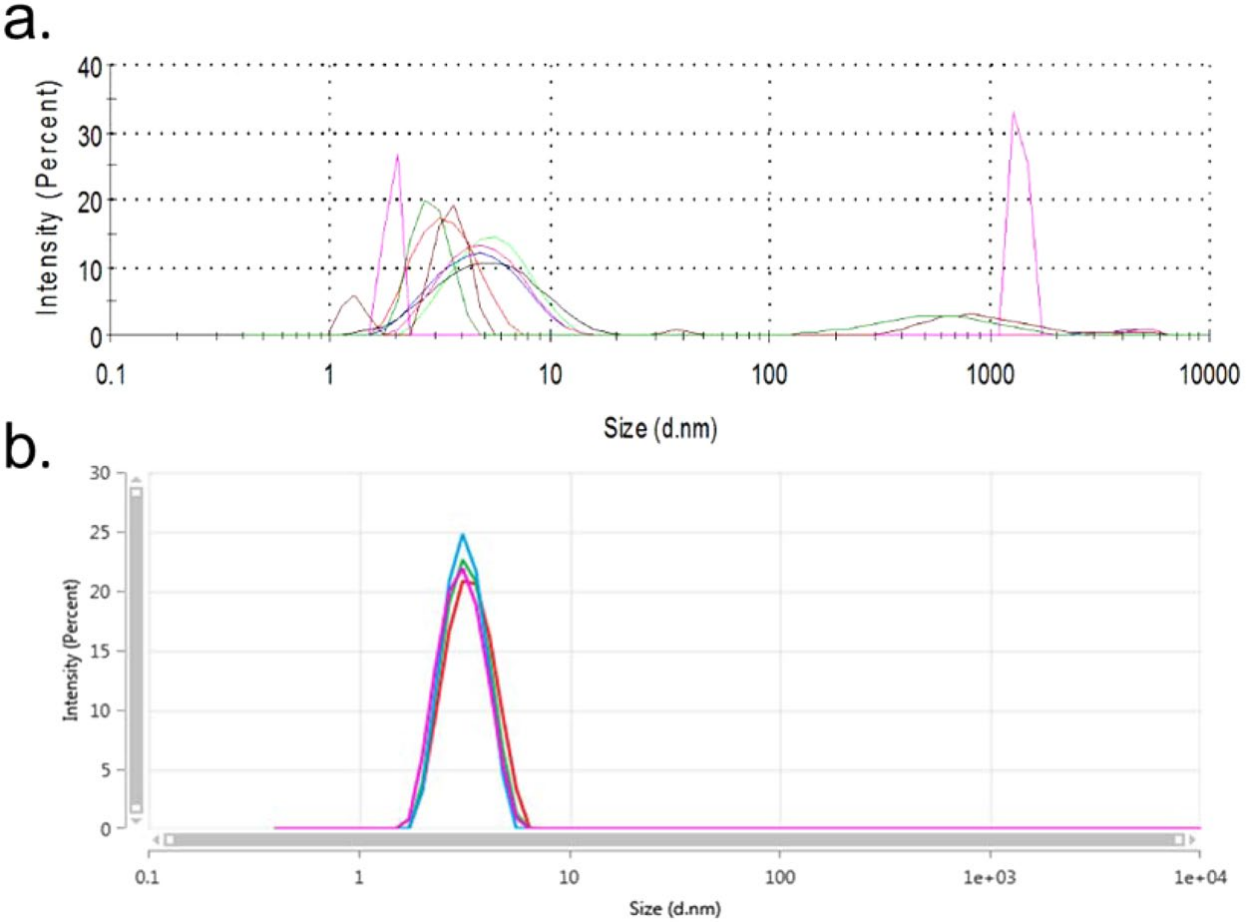
Software reported steady state intensity weighted particle size distributions measured for a thermally agitated 1 mg/ml dispersion of lysozyme, showing results for replicate measurements of the same aliquot of sample, measured using a traditional ‘dust rejection’ measurement process (**a**) and Adaptive Correlation (**b**). Replicate measurements in the top figure show highly variable results with broad peaks observed at a range of mode sizes due to skewing caused by trace amounts of aggregate material, whereas the same sample measured using Adaptive Correlation is significantly more precise and accurate.

This approach means that DLS data can be much more reproducible, allowing primary particle sizes to be accurately and confidently determined and the need for repeat measurements or a reliance on orthogonal techniques can be greatly reduced. This is demonstrated further in Fig. 8, where replicate measurements of the same sample of aggregated lysozyme produce accurate and repeatable results using the new Adaptive Correlation scheme, in comparison to the near pathological results from a traditional, count-rate, based dust-rejection method. Whilst the approach has been demonstrated in the use of particle size characterisation, any other measurement derived from DLS may benefit from this approach, including measurements of *k*_*D*_ and micro-rheology.

The approach may also be applied to other light scattering methods including but not limited to static light scattering for measurement of *A*_2_ and *M*_*w*_, and laser doppler velocimetry measurements for calculating properties such as electrophoretic mobility & zeta potential and, magnetisation and magnetic susceptibility.

## Conclusions

A new method of recording and processing data from DLS measurements has been demonstrated that improves the precision and accuracy of the reported particle size information. By individually correlating over a plurality of short sub measurements noise artefacts in the correlation function are reduced and the results of the fitting and inversion methods yield fewer variable results. Furthermore, performing a size analysis on each individual sub measurement and using an outlier identification method on the resultant *PdI*, we can produce data sets that accurately describe the steady state and transient components of the sample, each with improved accuracy and precision. This statistical approach means that the identification of transients is sample specific and not, for instance, based on a fixed size range rejection method which would require *a priori* knowledge of the sample. The resulting particle size distributions are therefore more representative of the sample regardless of whether they are monomodal, multimodal or polydisperse. Measurement times are also reduced by recording data until the statistics of the sample are suitably captured, meaning that for a well prepared and stable sample, repeat measurements can be performed in 24 s, or 1/5^th^ of the measurement time of some previously used methods.

Additional measurement optimisation has been demonstrated for the characterisation of low scattering samples, with an automatic increase in sub measurement duration being used to improve signal to noise in the measured correlation function. The improvements given by the new algorithm are demonstrated using dispersions of protein and polystyrene latex standards of a range of sizes, including polydisperse mixtures, and is applicable to any DLS measurement, including other properties derived from a DLS measurement including microrheology, as well as similar principles being applicable to static light scattering.

Whilst fractionation techniques such as HPLC used in conjunction with DLS offer better separation and resolution of different size components in dispersed systems, the non-invasive nature, low volume requirements and ease of measurement of batch DLS make it a preferable characterisation method in many cases, and the new Adaptive Correlation approach solves a primary criticism of the technique.

## Methods

All measurements were recorded using a Zetasizer Ultra (Malvern Panalytical Ltd, UK), fitted with a 10 mW 632.8 nm laser, with measurements performed using Non-invasive Back Scatter (NIBS)^33^ with a scattering angle of 173° in air and at a 90° scattering angle. Some comparative measurements were also performed using the Zetasizer Nano ZSP, with measurements performed with a scattering angle of 173^o^ in air. Samples include NIST traceable polystyrene latex colloids in a range of sizes, suspended in 10 mM NaCl solution, and Hen’s egg lysozyme, suspended in a pH 4.0 Citrate buffer, with both dispersants prepared using 200 nm filtered (Fisher brand, nylon) ultrapure water (18.2 MΩ). All materials were purchased from Sigma-Aldrich (UK) except the latices, which were purchased from Thermo-Scientific (US).

Dust was simulated using thermally agitated lysozyme, comprising a polydisperse aggregate component, and a polydisperse mixture of NIST traceable latex spheres, ranging in size between 100 nm and 8 µm in diameter (see supplementary information).
