# Author Correction: Improved Dynamic Light Scattering using an adaptive and statistically driven time resolved treatment of correlation data

**DOI:** 10.1038/s41598-024-51806-0

**Published:** 2024-01-23

**Authors:** Alexander V. Malm, Jason C. W. Corbett

**Affiliations:** Malvern Panalytical Ltd, Grovewood Rd, Malvern, Worcestershire, WR14 1XZ UK

Correction to: *Scientific Reports* 10.1038/s41598-019-50077-4, published online 18 September 2019.

This Article contains errors. The Supplementary Information file, which was included with the initial submission, was omitted from the original version of this Article. The Supplementary Information is now linked to this correction notice.

### Supplementary Information

Supplementary Information.

