# Supplementary material for: Author Correction: Improved Dynamic Light Scattering using an adaptive and statistically driven time resolved treatment of correlation data
Source: Sci Rep. 2024 Jan 23;14:2021. doi: 10.1038/s41598-024-51806-0 (PMC10805773; doi:10.1038/s41598-024-51806-0)
Supplement: Supplementary file 1 — Supplementary Information. [file 41598_2024_51806_MOESM1_ESM.pdf]

Supplementary information

Throughout the main manuscript, average particle size (Z_{Ave}), and particle size distributions are reported for samples containing contaminants. In order to demonstrate the performance of the Adaptive Correlation measurement procedure, controlled contributions of “dust” were required. Several sources of contaminants were studied, including material shed from syringe filters, airborne particulates, and dirty labware. Whilst the individual particle size distributions varied between these different sources, detectable particle sizes typically ranged from approximately 100nm to several microns. A polydisperse dust simulant was therefore formulated using a mixture of NIST traceable polystyrene latex spheres, dispersed in 10mM NaCl, ranging in size from 100nm to 8 µm in size. A particle size distribution for this mixture is shown in Figure S1. This mixture was diluted to an appropriate concentration such that the frequency of spikes in the detected scattering from this mixture was comparable to that for an isolated example of real contaminants, Figure S2.

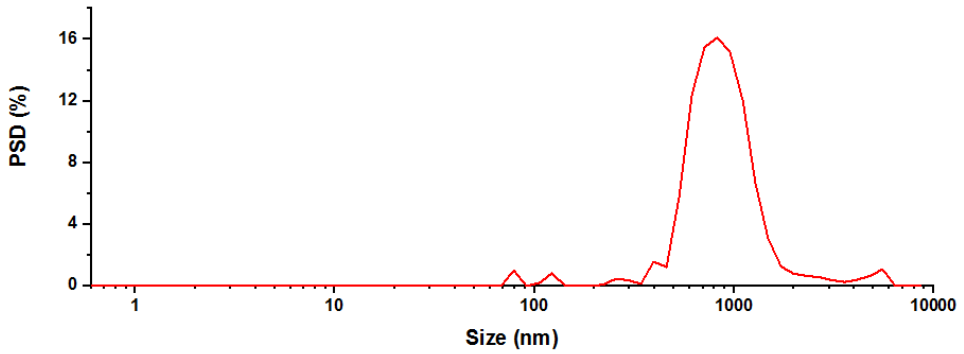

Figure S1: Intensity weighted particle size distribution for a mixture of NIST traceable latex spheres, used within the main text as a simulant for dust to dope other samples in a controlled manner.

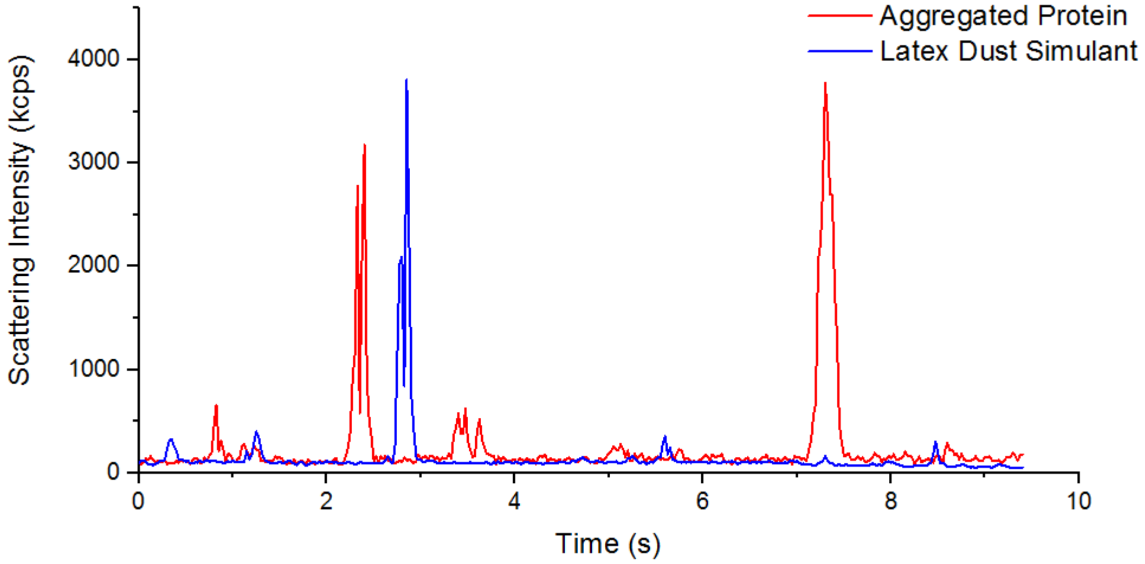

Figure S2: Scattering intensity measured in kilo-counts per second, as a function of time, for a back-scatter measurement of an aggregated protein sample and a dilute latex mixture. The latex mixture was diluted such that the frequency and magnitude of scattering intensity spikes was approximately comparable.

Where protein samples were used, the latex dust simulant was not appropriate as the latex spheres displayed affinity with the dispersed protein. Contaminants were introduced to protein dispersions in the form of protein aggregates, which are typically detectable by DLS in sizes ranging from around 50 nm to several microns, Figure S3.

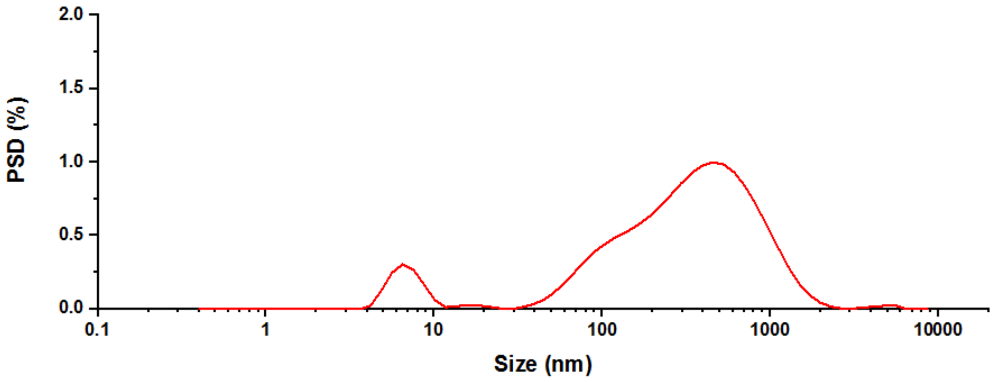

Figure S3: Intensity weighted particle size distribution for an aggregated protein sample, showing a detectable presence of large particles ranging from around 50nm to 5 µm.

Figure 2a of the main text shows the Z_{Ave} and *PdI* for a series of measurements of a 60 nm latex, with data processed as 10 s and 1 s duration correlation measurements, as well as results for averaged sets of 1 s correlations. The distributions of these results show that averaging the correlation functions of 10 1 s sub measurements gives better repeatability than correlating these as single 10s data sets. Figure S4 shows similar statistics can be observed over a range of differently sized particle dispersions.

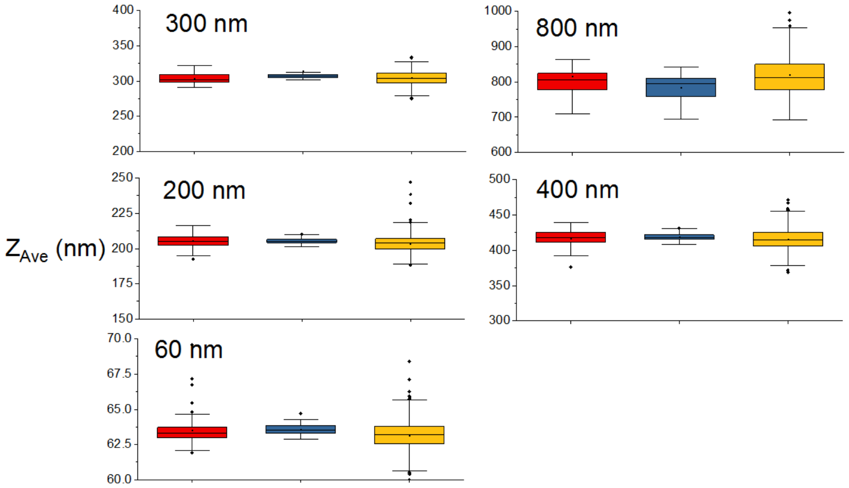

Figure S4: Distributions of ZAve measured for repeat measurements of polystyrene latex particles of a range of sizes. Results are shown for 10 s length measurements (red), individual 1 s measurements (yellow), and 1 s sub measurements, where the correlation functions are averaged in groups of 10 (blue). In all instances, the distributions of shorter individual sub measurements had more outliers, however the standard deviation is significantly reduced when several of these results are averaged.

In Section 2.4, the optimisation of sub measurement length for samples that display low scattering was discussed. Measurements of such a sample could be performed at a number of different sub measurement lengths, and the data quality compared, however this may result in lengthy method development procedures, which in turn may be complicated by time sensitivity of some samples.

An automated optimisation is therefore implemented whereby the sub measurement length is increased where required for an optimal number of photons to be detected during a sub measurement. By recording repeat measurements using a range of different sub measurement lengths, the repeatability of measurements for a range of different low scattering samples was

assessed. At increased sub measurement length, the noise in the correlation function approaches a limit whereby the acquisition of addition data provides no further improvement to the measurement, and as such, the repeatability of the measurements reaches a plateau, Figure S5. The optimal sub measurement length was therefore defined as the shortest sub measurement length that gave a minimal measurement standard deviation.

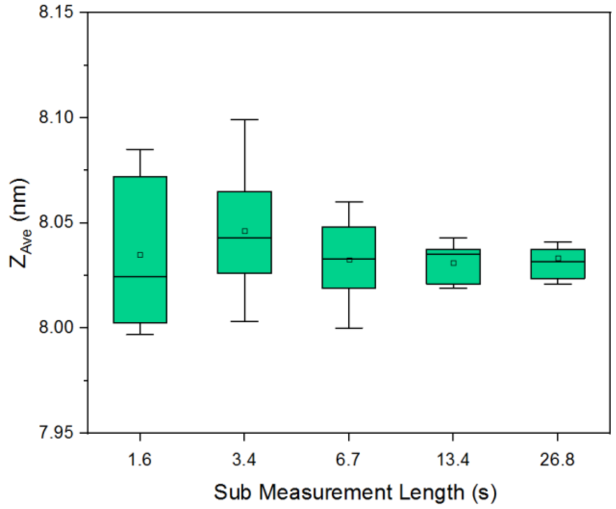

Figure S5: Distribution of measured Z_{Ave} for repeat measurements of a sample of 1 mg/mL BSA dispersed in 10 mM NaCl, recorded using different sub measurement times, showing a reduction in standard deviation for sub measurement lengths greater than 13.4 s.

Consolidating this data for measurements of different samples shows a common relationship between count rate and optimal sub measurement length, Figure S6. This allows a threshold number of photons per sub measurement to be derived, which was derived here to be 165k. This figure is likely coupled to both the architecture of the correlator used to perform these measurements and the optical properties of the scattering detection and the illuminating laser.

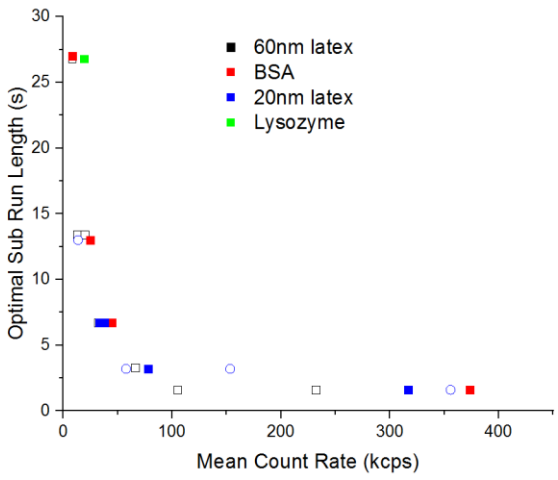

Figure S6: Optimal sub measurement (run) length in seconds as a function of mean count rate for measurements of a range of samples that displayed low scattering by serial dilution or attenuation of the detected scattering. Filled and hollow symbols represent measurements carried out using a 90º and 173º detection angle respectively.

To avoid normalisation errors occurring during a measurement, the sub measurement lengths are coupled to the correlator architecture such that sub measurements corresponding to the end of correlator groups can be used.

Figure 4b of the main text showed measurements performed with different sub measurement lengths for a sample of lysozyme, with an apparent large size component only observed with a short sub measurement length. Analysis of the correlation function baseline revealed temporally resolved noise present in the data recorded for short sub measurements. An alternative strategy to supress this noise may be to record a greater number of sub measurements, as described in Section 2.2.

Figure S7 however shows that whilst an increase in sub measurement length can supress this noise observed in low scattering samples, measurement of additional sub measurements does not give any reduction, and artefact size components may still be observed in distribution analysis, Figure S8.

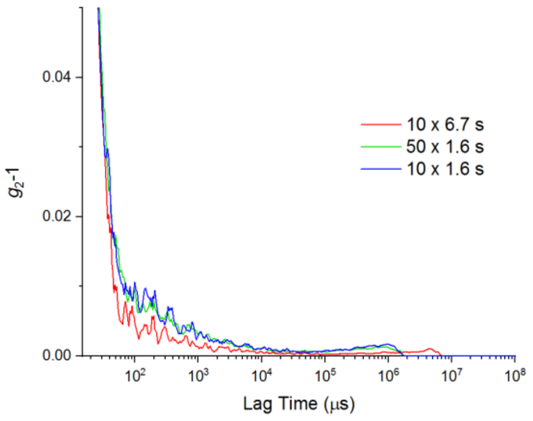

Figure S7: Correlation function baselines for measurements of a 1 mg/mL dispersion of lysozyme measured at a 90° detection angle, with data captured using different numbers of different length sub measurements. Increasing the number of sub measurements does not reduce the temporally resolved noise between 10^2 and 10^3 µs, whereas the measurements with an extended sub measurement length shows less noise.

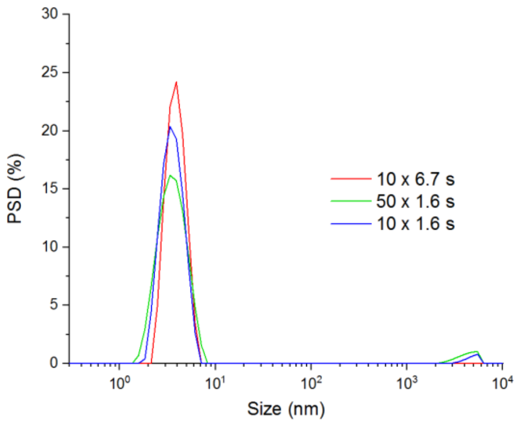

Figure S8: Intensity weighted particle size distributions for the sample of 1 mg/ml lysozyme measured in Figure S5. Both measurements with short sub measurements show an apparent large size component of the sample at around 5 µm, whereas the measurement with the extended sub measurement length does not show this component.

In a DLS measurement, at particles sizes > ~100 nm, light scattering intensity becomes increasingly less isotropic, with larger particles scattering more light at smaller scattering angles. If the large size components observed in Figure 4b of the main text and Figure S8 above were real, we would expect to detect these particles with greater intensity for a forward scattering DLS measurement. Figure S9 shows the particle size distribution for the same sample of 1 mg/mL lysozyme, which again shows that the sample is monomodal, and confirming that the large size peak in the data measured at 90° is an artefact.

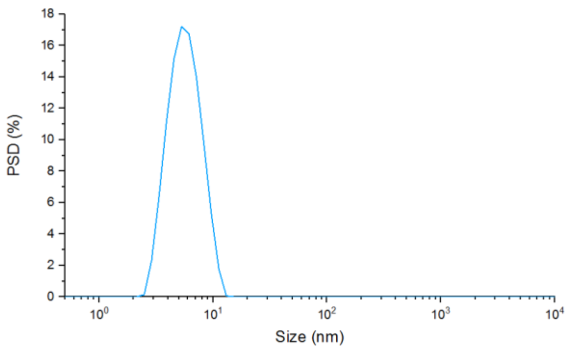

Figure S9: Intensity weighted particle size distribution for the sample of 1 mg/mL lysozyme measured previously, but measured with a 13° degree scattering angle, showing only one size population to be present.
